# Supplementary material for: Wastewater surveillance of SARS-CoV-2 mutational profiles at a university and its surrounding community reveals a 20G outbreak on campus
Source: PLoS One. 2022 Apr 14;17(4):e0266407. doi: 10.1371/journal.pone.0266407 (PMC9009614; doi:10.1371/journal.pone.0266407)
Supplement: S1 Text — (DOCX) [file pone.0266407.s001.docx]

**S1 Text.**

*Reverse transcription quantitative PCR (RT-qPCR) methods*

The RT-qPCR reaction was set up using Luna Universal Probe One-Step RT-qPCR kit (New England Biolabs, Ipswich, MA, USA) and 2019-nCoV Research Use Only qPCR Probe Assay primer/probe mix (Integrated DNA Technologies, Coralville, IA, USA). To minimize manual pipetting errors, the assay plate was prepared using the QIAgility automated PCR setup and liquid handling robot (Qiagen). The reaction mix (20 μL) consisted of 1X Reaction Mix, 1X RT Enzyme Mix, 500nM/125nM IDT 2019-nCoV Combined Primer/Probe Mix, and 2.5µL of nuclease-free water. The N2 primer/probe mix targeted a specific region of the SARS-CoV-2 nucleocapsid (N) gene. The RNA samples were diluted to a 1:5 ratio, with 5 μL of sample used per reaction. For the standard curve construction, the positive control, 2019-nCoV_N plasmid, (200,000 cp/μL) was linearized using ScaI-HF (New England Biolabs, Ipswich, MA, USA) and diluted from 2 x 105 cp/µL to 25 cp/µL. The RT-qPCR assay was amplified in the Applied Biosystem 7900HT Fast Real-Time PCR System with thermal cycling conditions as follows: reverse transcription for 10 min at 55 °C, initial denaturation for 1 min at 95°C, and 45 cycles of denaturation for 10s at 95 °C and extension for 1 min at 60 °C.
