# Supplementary material for: Wastewater surveillance of SARS-CoV-2 mutational profiles at a university and its surrounding community reveals a 20G outbreak on campus
Source: PLoS One. 2022 Apr 14;17(4):e0266407. doi: 10.1371/journal.pone.0266407 (PMC9009614; doi:10.1371/journal.pone.0266407)
Supplement: S1 Table — WW = wastewater. Samples with less than 50% coverage that are highlighted in gray were not included in the heatmap or co-occurrence analysis. (DOCX) [file pone.0266407.s002.docx]

**S1 Table.** Columbia WWTP influent samples used in this study and sequencing depth and coverage per barcode. WW=wastewater. Samples with less than 50% coverage that are highlighted in gray were not included in the heatmap or co-occurrence analysis.

| **Influent Sample** | **SARS-CoV-2 copies x 10^4^/L WW** | **Average Depth** | **Coverage [%]** |
| --- | --- | --- | --- |
| 7/19/20 | 2.17 | 33.5 | 34.0 |
| 7/22/20 | 6.25 | 167 | 64.0 |
| 8/2/20 | 0.70 | 47.3 | 32.1 |
| 8/5/20 | 1.09 | 138 | 52.5 |
| 8/9/20 | 0.87 | 152 | 66.5 |
| 8/12/20 | 1.67 | 225 | 73.2 |
| 9/2/20 | 11.4 | 159 | 49.6 |
| 9/6/20 | 4.28 | 210 | 70.8 |
| 9/13/20 | 2.87 | 117 | 41.3 |
| 9/16/20 | 3.36 | 161 | 51.8 |
| 9/20/20 | 1.65 | 233 | 74.8 |
| 9/23/20 | 1.85 | 42.3 | 19.9 |
| 9/27/20 | 1.99 | 60.4 | 20.7 |
| 9/30/20 | 2.01 | 128 | 35.2 |
