# Supplementary material for: Wastewater surveillance of SARS-CoV-2 mutational profiles at a university and its surrounding community reveals a 20G outbreak on campus
Source: PLoS One. 2022 Apr 14;17(4):e0266407. doi: 10.1371/journal.pone.0266407 (PMC9009614; doi:10.1371/journal.pone.0266407)
Supplement: S2 Table — WW = wastewater; UofSC = University of South Carolina. (DOCX) [file pone.0266407.s003.docx]

**S2 Table.** University of South Carolina campus samples used in this study and sequencing depth and coverage per barcode. WW=wastewater; UofSC=University of South Carolina.

| **UofSC Sample** | **SARS-CoV-2 copies x 10^4^/L WW** | **Average Depth** | **Coverage [%]** |
| --- | --- | --- | --- |
| Site 1 8/28/20 | 445 | 377 | 99.8 |
| Site 8 8/28/20 | 947 | 379 | 99.8 |
| Site 5 9/4/21 | 237 | 412 | 99.8 |
| Site 11 9/4/20 | 268 | 410 | 99.8 |
| Site 1 9/11/20 | 278 | 318 | 96.7 |
