# Supplementary material for: Wastewater surveillance of SARS-CoV-2 mutational profiles at a university and its surrounding community reveals a 20G outbreak on campus
Source: PLoS One. 2022 Apr 14;17(4):e0266407. doi: 10.1371/journal.pone.0266407 (PMC9009614; doi:10.1371/journal.pone.0266407)
Supplement: S3 Table — (DOCX) [file pone.0266407.s004.docx]

**S3 Table.** Concurrent mutations identified in wastewater samples that were validated with GESS [1] on December 22, 2021.

| **Mutation 1** | **Mutation 2** | **Count** | **Concurrence Ratio (%)** |
| --- | --- | --- | --- |
| 10,319 c > t | 1358 g > a | 745 | 95.6 |
| 10,319 c > t | 14,805 c > t | 23,951 | 88.9 |
| 12,473 c > t | 1358 g > a | 745 | 90.0 |
| 12,473 c > t | 14,805 c > t | 23,951 | 53.9 |
| 12,473 c > t | 18,424 a > g | 52,779 | 60.9 |
| 12,473 c > t | 3037 c > t | 308,335 | 99.2 |
| 12,473 c > t | 8083 g > a | 21,423 | 53.7 |
| 13,201 g > t | 1358 g > a | 745 | 95.0 |
| 13,201 g > t | 14,805 c > t | 23,951 | 75.3 |
| 13,201 g > t | 18,424 a > g | 52,779 | 79.7 |
| 13,201 g > t | 3037 c > t | 308,335 | 99.5 |
| 13,201 g > t | 8083 g > a | 21,423 | 74.6 |
| 1358 g > a | 16,748 a > g | 880 | 95.1 |
| 1358 g > a | 25,907 g > t | 52,434 | 94.7 |
| 1358 g > a | 27,964 c > t | 57,669 | 96.6 |
| 1358 g > a | 28,472 c > t | 52,961 | 95.4 |
| 1358 g > a | 28,869 c > t | 58,037 | 96.7 |
| 14,408 c > t | 241 c > t | 306,949 | 99.4 |
| 14,805 c > t | 16,748 a > g | 880 | 99.3 |
| 14,805 c > t | 25,907 g > t | 52,434 | 88.2 |
| 14,805 c > t | 27,964 c > t | 57,669 | 88.8 |
| 14,805 c > t | 28,472 c > t | 52,961 | 88.4 |
| 14,805 c > t | 28,869 c > t | 58,037 | 88.8 |
| 16,748 a > g | 18,424 a > g | 52,779 | 99.3 |
| 16,748 a > g | 3037 c > t | 308,335 | 99.5 |
| 16,748 a > g | 8083 g > a | 21,423 | 92.5 |
| 18,424 a > g | 25,907 g > t | 52,434 | 98.8 |
| 18,424 a > g | 27,964 c > t | 57,669 | 98.8 |
| 18,424 a > g | 28,472 c > t | 52,961 | 98.6 |
| 18,424 a > g | 28,869 c > t | 58,037 | 98.8 |
| 25,907 g > t | 3037 c > t | 308,335 | 99.8 |
| 25,907 g > t | 8083 g > a | 21,423 | 98.7 |
| 27,964 c > t | 3037 c > t | 308,335 | 99.7 |
| 27,964 c > t | 8083 g > a | 21,423 | 99.2 |
| 28,472 c > t | 3037 c > t | 308,335 | 99.8 |
| 28,472 c > t | 8083 g > a | 21,423 | 98.8 |
| 28,869 c > t | 3037 c > t | 308,335 | 99.7 |
| 28,869 c > t | 8083 g > a | 308,335 | 99.2 |
