# Supplementary figures and images for: Wastewater surveillance of SARS-CoV-2 mutational profiles at a university and its surrounding community reveals a 20G outbreak on campus
Source: PLoS One. 2022 Apr 14;17(4):e0266407. doi: 10.1371/journal.pone.0266407 (PMC9009614; doi:10.1371/journal.pone.0266407)

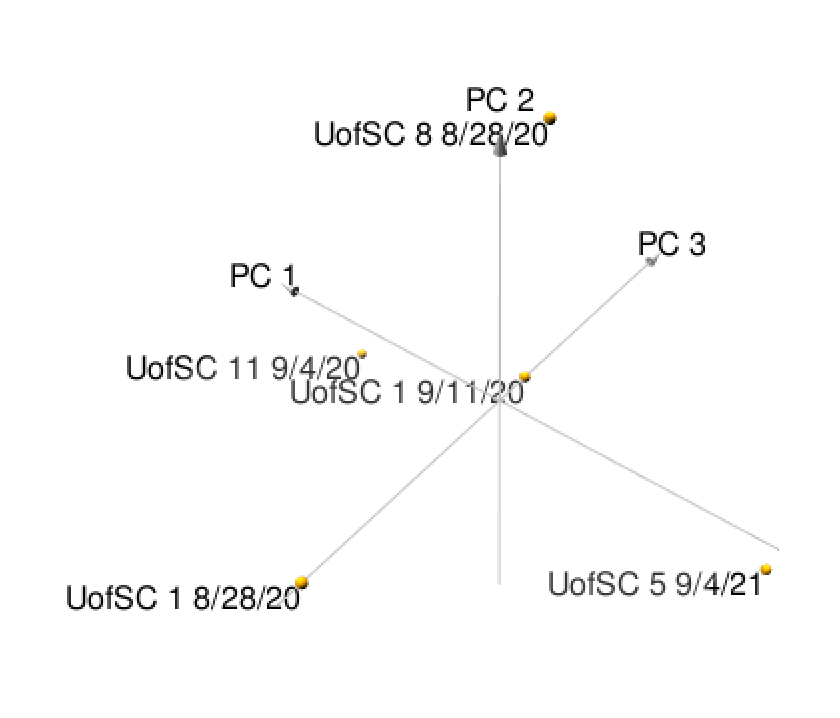

Supplement: S1 Fig — (TIF) [file pone.0266407.s005.tif]

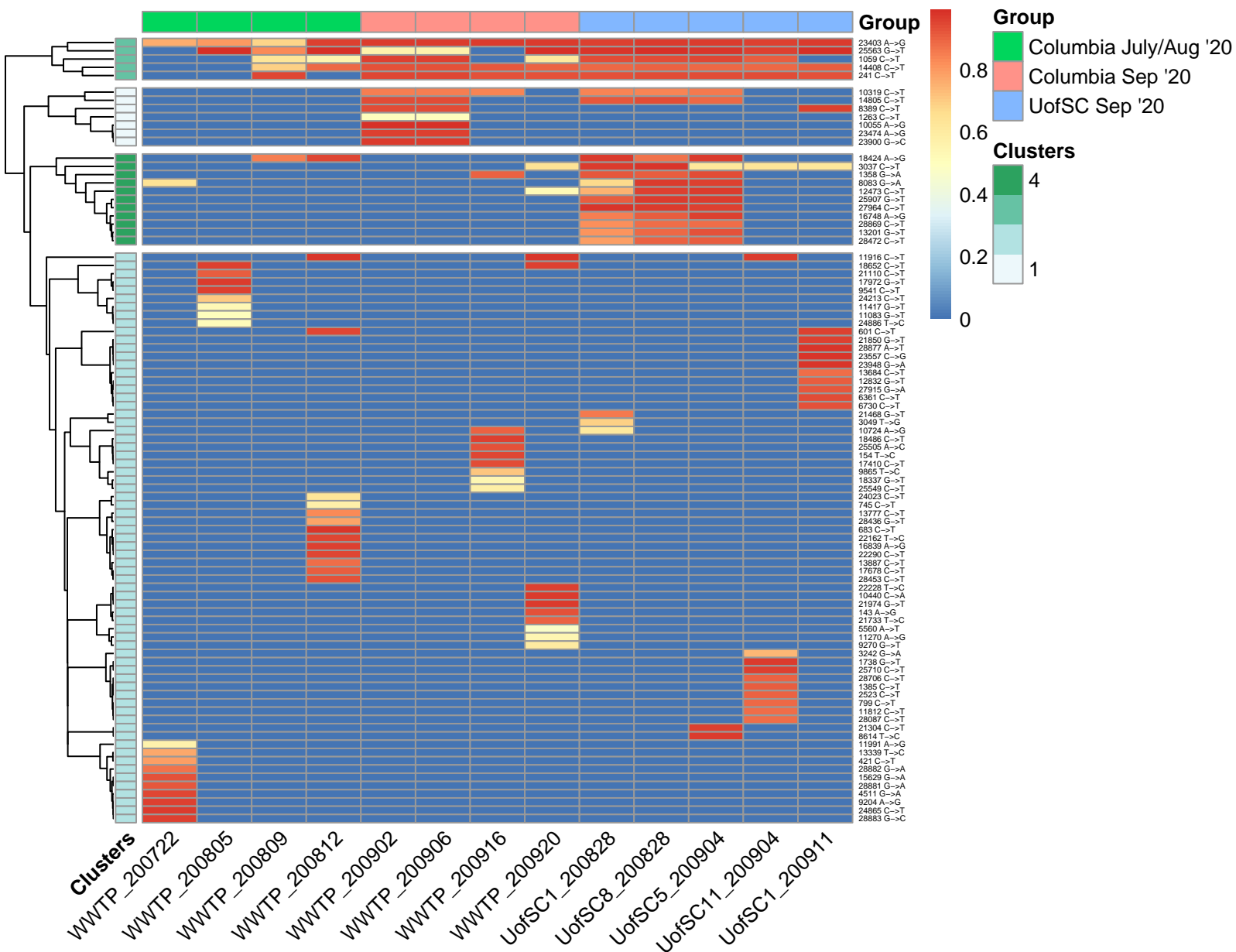

Supplement: S2 Fig — (PDF) [file pone.0266407.s006.pdf]
