# Supplementary material for: Wastewater surveillance of SARS-CoV-2 mutational profiles at a university and its surrounding community reveals a 20G outbreak on campus
Source: PLoS One. 2022 Apr 14;17(4):e0266407. doi: 10.1371/journal.pone.0266407 (PMC9009614; doi:10.1371/journal.pone.0266407)
Supplement: Attachment — Submitted filename: review.docx [file pone.0266407.s007.docx]

The authors report and compare the sequence diversity of SARS-CoV-2 from wastewater influent to the Columbia, South Carolina, metropolitan wastewater treatment plant (WWTP) and four sites from the University of South Carolina (UofSC) campus during July-September 2020. In this study, 154 unique mutations were detected across all samples; of those 26 mutations were shared between the university and the WWTP. Their main conclusions are:	1- there is a close relationship between the prevalent SARS-CoV-2 genotypes from the UofSC campus and the Columbia WWTP.

2- wastewater surveillance at the building level at a university captures sequence diversity not detected in the WWTP, thus offering a balance between WWTPs and clinical sequencing.

It is important to track and characterize mutations at large volume facilities as well as obtain a higher resolution at a local level, which is much closer to the potential source. Such methods when employed systematically can help in deploying mitigating efforts to contain outbreaks. Surveillance, quantification, sequencing, and comparison of results is intricate as wastewater is a complex matrix. Additionally, the impact of temporal and geographical variability needs to be carefully assessed for data from wastewater surveillance to be meaningful. Therefore, this comparison study is valuable and important. However, there are concerns regarding the methodology and the conclusions derived from the results, which need to be addressed before the paper can be published.

Major issues

1- Lines 227-228. To compare the read frequencies differences between the WWTP influent collected in July and August, the WWTP influent collected in September, and the university wastewater samples collected in September, the authors use the student’s t-test. The t-test is generally used for pairwise comparisons, for comparing three or more conditions, ANOVA for multiple comparisons is more suitable. The authors should use ANOVA and assess the significance of the differences detected.
2- Another limitation that should be addressed is that at low RNA copy numbers, the read frequencies cannot be correlated as absolute frequencies of mutations found in a sample. The aliquots used for cDNA synthesis will show stochastic variations due to the low RNA copy numbers. This results in biased PCR amplifications and the read frequencies might vary among the replicates from the same sample. Therefore, at low copy numbers (as seen in the Columbia WWTP), comparing read frequencies may not be appropriate.
3- These caveats are also problematic as read frequencies were used for the probabilistic models in the co-occurrence and linkage analysis. The methodologies used for the co-occurrence and linkage analysis should be further detailed in the paper.
4- The use of the terms co-occurrent and concurrent should be explained in more detail. For example, in line 293, “here” implies that in other instances co-occurring has another meaning? Additionally, the authors should explain why none of the pairs of mutations identified by linkage overlapped with those identified through the R package cooccur.

Minor issues

1- More precise information would be useful for Figure 1. What were the cases of isolation or quarantine based on? Did they test positive for SARS-CoV-2 or as suggested by the legend suspected of exposure?
2- Line 170- Figure 2 is mentioned to visualize site 1 but Figure 4, panel B should be referenced to show the overlapping points. The authors use the PCA analysis showing overlapping points for the UofSC site 1 for August 28 and September 11 to suggest sequence stability. The average shedding time of the SARS-CoV-2 virus is two weeks, the same time lapse between the two sampling events at site 1. Therefore, the authors should explain the sequence stability because of the temporal and spatial distribution of the samples.
3- Figure 4. The color coding of the groups has been switched on panel A. Red should be assigned to Sept and blue to July/Aug.
4- Line 287. Something seems to be missing. “complementary techniques to identify co-occurrence. patterns in sequence space.”
