# Supplementary material for: Wastewater surveillance of SARS-CoV-2 mutational profiles at a university and its surrounding community reveals a 20G outbreak on campus
Source: PLoS One. 2022 Apr 14;17(4):e0266407. doi: 10.1371/journal.pone.0266407 (PMC9009614; doi:10.1371/journal.pone.0266407)
Supplement: Attachment — Submitted filename: plos1_wwtp_reviewer_notes.docx [file pone.0266407.s008.docx]

In the submitted manuscript “Wastewater surveillance illustrates overlapping SARS-CoV-2 mutational profiles between a university campus and its surrounding community”, the authors present the results of municipal-level wastewater treatment plant (WWTP) surveillance and campus-level surveillance in concert. They identify common trends and also differences between the WWTP and campus samples along with presentation of results of a variety of statistical tools that aim to deconvolute the wastewater mixtures into co-occurring genotypes. I have a few concerns and suggestions listed below:

*1) Interpretation of the SNPs over time (Page 10-12):* The co-occurrence result is challenging to interpret, especially the linkage results on page 13. I like Figure 6 A and B, and think they should stay in some form (and maybe even largely as-is), but they need more interpretation. What viral context do these networks reveal?

The authors state on page 10 (lines 209-221) that Cluster 4 mutations, including 25563 g>t, are found in all samples at close to 100% frequency, which would suggest that at the time(s) of sampling, nearly all detectable SARS-CoV-2 in Columbia and UofSC are of the 20C clade (or a descendant). However, one of the other major markers for clade 20C, 1059 c>t, is found in fewer enough samples that it is grouped into the “all conditions, albeit less consistently” Cluster 3. To my eye, the differences between 1059 and 25563 are not huge in the campus samples. All but one (site 1, 9/11) have both. From this we might surmise a strong presence of 20C (or descendant) on the campus at the time of sampling. The less consistent signal in the WWTP samples suggest a more complicated mix of genotypes in the broader area.

Alternatively, if we expect to find 1059 in all samples where the 25563 mutant is found, might that imperfect correspondence be the result of wastewater sequencing itself, with this more challenging substrate producing more piecemeal results as one of the sensitivity tradeoffs? Along these same lines of sensitivity, I note that there is no discussion of the spike mutation D614G (23403 a>g). Do the V3 amplicons poorly amplify this region? Can the authors speculate on the reasons?

*2) A 20G outbreak on campus:* I think at least one of the things the authors have revealed (Networks in Fig 6, SNPs in Fig 5/ S1, and lists thereof on pages 10-12), is **it looks like they caught a 20G outbreak in-progress on campus.** Hints of which are seen in the WWTP, but not nearly as consistently as the 20G signal caught in-the-act on campus. The authors should emphasize this finding/interpretation, even as it may require re-writing of the co-occurrence results.

On lines 230-232, the authors describe a significance test whereby 6 mutations were different between September WWTP and University samples “1358g > a, 3037c > t 13,201g > t, 18,424a > g, 25,907g > t, and 27,964c > t.” Of these, 3037 c >t is hard to explain (it is found in all 20A and all descendants, along with 241, 14408, and 23403), but 18424, 25907, and 27964 are some of the markers of 20G. If these 3 are all going in the same direction (are they?), they may be evidence that either the University or the City were undergoing a wave of 20G that the other was not (<https://github.com/nextstrain/ncov/blob/master/defaults/clades.tsv>).

The tests continue on lines 232-236, with summer City samples compared to University samples. Two more 20G markers make this list: 10319 and 28472. Another universal 20A marker does too (241 c>t). I think the interpretations of each are similar to those on the previous tests.

Together, the above, and knowing the global context of mutations 10319, 18424, 25907, 27964, and 28472 as markers of 20G, the authors may have bona-fide evidence of a 20G outbreak in-progress on this campus. While less consistent hints of 20G are found in the City samples, the 20G signal is quite “bright” in 3 of the campus samples (Fig S1), (Site 1 8/28, Site 8 8/28, Site 5 9/4) and absent in (Site 11 9/4, Site 1 9/11).

*3) PCA plots in 4B:* The PCA in Figure 4B does not seem to agree with the other findings of sample similarity/dissimilarity. The two samples in the center are unlikely to be that similar.

I find it hard to come up with a scenario in which two samples would completely overlap on a PCA plot unless they were identical (UofSC points 1 8/28/20 and 1 9/11/20). I guess it could be possible if their differences only showed up on an N-th PC and not PC1 or 2? At minimum, the authors should check their input matrix/dataframe for the PCA in 4B, verify whether these two datapoints are identical or not, and add/expand comments on this to help explain. Figure 4’s legend needs to also explain what is being plotted, not just “samples”. Is it a singular inferred genotype per sample or a species proportion?

Moreover, regardless of what interpretation of genotype(s) is being computed for a dissimilarity score, it is difficult to believe that these two samples in particular would be perfectly identical on the first two PCs, given how many SNPs they have not-in-common, per figure 5 Clusters 3,2, and 1.

***Other issues***

*Introduction*

The need to have Figure 5 and Figure S1 which only differ by the S1 having text labels on each row is odd? Figure S1 seems to be sufficient, and an idea would be to merge the two figures back into one, and in the body of the article.

Line 42-43: The statement that “reinfection with SARS-CoV-2 has been demonstrated to occur on college campuses in at least 16% of students” is interpreted incorrectly from Reference #8. The estimated reinfection rate in Reference 8 was 2.2% with 16% of the population susceptible to reinfection. This needs to be fixed.

*Figure 1*

Figure 1’s X axis makes sense in set notation, but probably not as what most readers will expect from a scientific figure. Would it be appropriate to replace the current x-axis label with the latter date of each given set’s range?

*Figure 3*

Fig 3A is challenging to interpret. The gray dots along the axes aren’t easy to mentally place alongside the colorful diagonal dots. I advise the authors to rework this figure to 3 horizontal graphs, such as a top level with the gray UofSC-only sites, a middle with the colorful shared sites, and a bottom level with Columbia WWTP-only sites. Dot jitter could be used for legibility. Also, the Y-axis on Fig 3A uses the abbreviation “USC” not found elsewhere in the manuscript.

*Figure 4*

Axis labels and legends lack capitalization. Name the 4A legend something like “WWTP groups.”

*Linkage results and discussion*

The linkage discussion on divergence on p 13 is incomplete and could be made more clear. What is the significance or lack thereof of the disagreements? Also, there are some typos on line 293.

*Other minor notes*

On line 78, MGD has not been defined.

On and around line 100, which native barcoding kit(s) were used? Expansion 1-12, 13-24, Expansion 96?

On line 104, unless all amplicons are exactly 400 bp, replace with “~400 bp.”

On line 113, was the flow cell R9.4? If possible, more specific is better.

Citation 16 only has author initials.
