# Supplementary material for: Wastewater surveillance of SARS-CoV-2 mutational profiles at a university and its surrounding community reveals a 20G outbreak on campus
Source: PLoS One. 2022 Apr 14;17(4):e0266407. doi: 10.1371/journal.pone.0266407 (PMC9009614; doi:10.1371/journal.pone.0266407)
Supplement: Attachment — Submitted filename: Response to Reviewers.docx [file pone.0266407.s009.docx]

We thank the reviewers for taking the time to review this manuscript and for their many insightful suggestions and comments. In the responses below, please refer to the revised manuscript with track changes on in order to find the referenced line numbers.

In the revised manuscript, we have removed the section on linkage analysis because the consensus from reviewers was that the section was confusing and did not add value to the manuscript. During the review process, we also discovered that the read filtering by inStrain was too stringent for long-read sequencing data (retaining only 13% of the sequencing reads) and unfortunately cannot be adjusted for variant calling. Since the analysis in this manuscript relies heavily on the read frequencies, the results are sensitive to the read filtering thresholds. We have communicated this issue to the inStrain team via GitHub. However, for the purposes of presenting the SARS-CoV-2 data to the scientific community in a timely manner, we chose to use the ARTIC minion program to call variants instead of inStrain profile. The methods section has been revised to reflect these changes (please refer to lines 136-140). The total number of mutations called by artic minion was less (92) compared to inStrain (154). These changes are reflected in the manuscript, as well as the figures. The major findings reported in the manuscript such as the importance of localized sampling and, as pointed out by Reviewer #2, an outbreak of the 20G clade on campus are unchanged.

**Reviewer #1**

The authors report and compare the sequence diversity of SARS-CoV-2 from wastewater influent to the Columbia, South Carolina, metropolitan wastewater treatment plant (WWTP) and four sites from the University of South Carolina (UofSC) campus during July-September 2020. In this study, 154 unique mutations were detected across all samples; of those 26 mutations were shared between the university and the WWTP. Their main conclusions are:

1- there is a close relationship between the prevalent SARS-CoV-2 genotypes from the UofSC campus and the Columbia WWTP.

2- wastewater surveillance at the building level at a university captures sequence diversity not detected in the WWTP, thus offering a balance between WWTPs and clinical sequencing.

It is important to track and characterize mutations at large volume facilities as well as obtain a higher resolution at a local level, which is much closer to the potential source. Such methods when employed systematically can help in deploying mitigating efforts to contain outbreaks. Surveillance, quantification, sequencing, and comparison of results is intricate as wastewater is a complex matrix. Additionally, the impact of temporal and geographical variability needs to be carefully assessed for data from wastewater surveillance to be meaningful. Therefore, this comparison study is valuable and important. However, there are concerns regarding the methodology and the conclusions derived from the results, which need to be addressed before the paper can be published.

Major issues

1- Lines 227-228. To compare the read frequencies differences between the WWTP influent collected in July and August, the WWTP influent collected in September, and the university wastewater samples collected in September, the authors use the student’s t-test. The t-test is generally used for pairwise comparisons, for comparing three or more conditions, ANOVA for multiple comparisons is more suitable. The authors should use ANOVA and assess the significance of the differences detected. Thank you for this suggestion. We have revised the manuscript to use an ANOVA and subsequent Tukey’s test. Please refer to Supplementary Dataset S1 and lines 148-149 and 269-273 in the manuscript.
2- Another limitation that should be addressed is that at low RNA copy numbers, the read frequencies cannot be correlated as absolute frequencies of mutations found in a sample. The aliquots used for cDNA synthesis will show stochastic variations due to the low RNA copy numbers. This results in biased PCR amplifications and the read frequencies might vary among the replicates from the same sample. Therefore, at low copy numbers (as seen in the Columbia WWTP), comparing read frequencies may not be appropriate.

Thank you for your comment. Although we agree that low copy number could be problematic, we are confident that the RNA concentrations in the WWTP influent were sufficient to avoid appreciable stochastic variations. We demonstrated in the PCA that SARS-CoV-2 sequences amplified from WWTP influent in July-September are largely consistent (Figure 4A), except for the earliest and latest data points (8/12/20 and 9/20/20) from this sample set. Furthermore, the heatmap in Figure 5 illustrates the reproducibility of the detected mutations, given that the mutational profiles of WWTP influent collected on 09/02/20 and 09/06/20 are nearly identical.

3- These caveats are also problematic as read frequencies were used for the probabilistic models in the co-occurrence and linkage analysis. The methodologies used for the co-occurrence and linkage analysis should be further detailed in the paper. Thank you for your comments. We have revised the section on co-occurrence for clarity (please see lines 304-308) and we have removed the linkage analysis section in favor of further discussion regarding a 20G outbreak on campus.
4- The use of the terms co-occurrent and concurrent should be explained in more detail. For example, in line 293, “here” implies that in other instances co-occurring has another meaning? Additionally, the authors should explain why none of the pairs of mutations identified by linkage overlapped with those identified through the R package cooccur.

Thank you for your comment. Since co-occurrence could refer to mutations identified from within the same viral genotype (as opposed to the same wastewater sample), line 293 (original manuscript) is intended to clarify to the reader that the mutations only arise within the same sample. We have revised the manuscript to further to clarify these points. Please refer to lines 304-308 in the revised manuscript. We have removed the section regarding linkage analysis because we felt that it detracted from the main story presented in the manuscript. Instead, we have incorporated additional discussion of the co-occurrence analysis and its significance in the detection of a 20G outbreak on campus lines 18-21, 247-251, 269-273, 308-310, and 317-319 of the revised manuscript).

Minor issues

1- More precise information would be useful for Figure 1. What were the cases of isolation or quarantine based on? Did they test positive for SARS-CoV-2 or as suggested by the legend suspected of exposure? Thank you for this observation. We have clarified that cases of isolation consider individuals who tested positive for COVID-19 whereas cases of quarantine refer to individuals with close contact to a confirmed case of COVID-19. Please see lines 75-76 in the revised manuscript.
2- Line 170- Figure 2 is mentioned to visualize site 1 but Figure 4, panel B should be referenced to show the overlapping points. The authors use the PCA analysis showing overlapping points for the UofSC site 1 for August 28 and September 11 to suggest sequence stability. The average shedding time of the SARS-CoV-2 virus is two weeks, the same time lapse between the two sampling events at site 1. Therefore, the authors should explain the sequence stability because of the temporal and spatial distribution of the samples.

Thank you for your comment. We found that in a 3D-PCA, the two data points are in fact not overlapping. We have added the 3D-PCA plot as a supplementary figure (Supplementary Figure S1 in the revised manuscript). We also added the reference to Figure 4, panel B. Please refer to lines 187-189 in the revised text.

3- Figure 4. The color coding of the groups has been switched on panel A. Red should be assigned to Sept and blue to July/Aug.

Thank you for this observation. We have made the correction to the figure.

4- Line 287. Something seems to be missing. “complementary techniques to identify co-occurrence. patterns in sequence space.” Thank you for this observation. The sentence should have read “Therefore, the results of these two different methods may be considered as complementary techniques to identify co-occurrence patterns in sequence space.” However, this sentence has been removed in the revised document because we have eliminated the section discussing linkage analysis in favor of further discussion of a 20G outbreak on campus.

**Reviewer #2**

In the submitted manuscript “Wastewater surveillance illustrates overlapping SARS-CoV-2 mutational profiles between a university campus and its surrounding community”, the authors present the results of municipal-level wastewater treatment plant (WWTP) surveillance and campus-level surveillance in concert. They identify common trends and also differences between the WWTP and campus samples along with presentation of results of a variety of statistical tools that aim to deconvolute the wastewater mixtures into co-occurring genotypes. I have a few concerns and suggestions listed below:

*1) Interpretation of the SNPs over time (Page 10-12):* The co-occurrence result is challenging to interpret, especially the linkage results on page 13. I like Figure 6 A and B, and think they should stay in some form (and maybe even largely as-is), but they need more interpretation. What viral context do these networks reveal?

Thank you for this suggestion. We have revised the manuscript to include additional interpretation of Figures 6A and 6B. Please see lines 314-319 in the revised text. We have eliminated the section on linkage analysis.

The authors state on page 10 (lines 209-221) that Cluster 4 mutations, including 25563 g>t, are found in all samples at close to 100% frequency, which would suggest that at the time(s) of sampling, nearly all detectable SARS-CoV-2 in Columbia and UofSC are of the 20C clade (or a descendant). However, one of the other major markers for clade 20C, 1059 c>t, is found in fewer enough samples that it is grouped into the “all conditions, albeit less consistently” Cluster 3. To my eye, the differences between 1059 and 25563 are not huge in the campus samples. All but one (site 1, 9/11) have both. From this we might surmise a strong presence of 20C (or descendant) on the campus at the time of sampling. The less consistent signal in the WWTP samples suggest a more complicated mix of genotypes in the broader area.

Alternatively, if we expect to find 1059 in all samples where the 25563 mutant is found, might that imperfect correspondence be the result of wastewater sequencing itself, with this more challenging substrate producing more piecemeal results as one of the sensitivity tradeoffs? Along these same lines of sensitivity, I note that there is no discussion of the spike mutation D614G (23403 a>g). Do the V3 amplicons poorly amplify this region? Can the authors speculate on the reasons?

Thank you for these observations. Based on your observation regarding the D614G mutation, we revisited the BAM files and found that this mutation was high frequency (>95%) in all of the university samples. However, overly stringent read filtering by inStrain had removed enough reads such that this mutation was below our 50x depth threshold. For this reason, we chose to rely on the nanopolish results from the artic minion pipeline for variant calling and read frequencies. The D614G mutation is represented in Figure 5 and Supplementary Figure S2 in all university samples.

*2) A 20G outbreak on campus:* I think at least one of the things the authors have revealed (Networks in Fig 6, SNPs in Fig 5/ S1, and lists thereof on pages 10-12), is **it looks like they caught a 20G outbreak in-progress on campus.** Hints of which are seen in the WWTP, but not nearly as consistently as the 20G signal caught in-the-act on campus. The authors should emphasize this finding/interpretation, even as it may require re-writing of the co-occurrence results.

Thank you for this suggestion. We agree that this is a significant finding and have included this interpretation in the revised manuscript (lines 18-21, 247-251, 269-273, 308-310, and 317-319, section headers, and manuscript title).

On lines 230-232, the authors describe a significance test whereby 6 mutations were different between September WWTP and University samples “1358g > a, 3037c > t 13,201g > t, 18,424a > g, 25,907g > t, and 27,964c > t.” Of these, 3037 c >t is hard to explain (it is found in all 20A and all descendants, along with 241, 14408, and 23403), but 18424, 25907, and 27964 are some of the markers of 20G. If these 3 are all going in the same direction (are they?), they may be evidence that either the University or the City were undergoing a wave of 20G that the other was not (<https://github.com/nextstrain/ncov/blob/master/defaults/clades.tsv>).

The tests continue on lines 232-236, with summer City samples compared to University samples. Two more 20G markers make this list: 10319 and 28472. Another universal 20A marker does too (241 c>t). I think the interpretations of each are similar to those on the previous tests.

Together, the above, and knowing the global context of mutations 10319, 18424, 25907, 27964, and 28472 as markers of 20G, the authors may have bona-fide evidence of a 20G outbreak in-progress on this campus. While less consistent hints of 20G are found in the City samples, the 20G signal is quite “bright” in 3 of the campus samples (Fig S1), (Site 1 8/28, Site 8 8/28, Site 5 9/4) and absent in (Site 11 9/4, Site 1 9/11).

We are grateful to the reviewer for these significant observations and have made several major modifications to the manuscript to incorporate these discussion points. Please refer to our response above.

*3) PCA plots in 4B:* The PCA in Figure 4B does not seem to agree with the other findings of sample similarity/dissimilarity. The two samples in the center are unlikely to be that similar.

I find it hard to come up with a scenario in which two samples would completely overlap on a PCA plot unless they were identical (UofSC points 1 8/28/20 and 1 9/11/20). I guess it could be possible if their differences only showed up on an N-th PC and not PC1 or 2? At minimum, the authors should check their input matrix/dataframe for the PCA in 4B, verify whether these two datapoints are identical or not, and add/expand comments on this to help explain. Figure 4’s legend needs to also explain what is being plotted, not just “samples”. Is it a singular inferred genotype per sample or a species proportion?

Thank you for your comments. We have checked the input matrix for accuracy. In fact, the pairwise comparisons for these two samples were not identical, although they were similar. However, in a 2D-PCA plot in which only 51.2% of the variance is explained by the two principal components, the data points representing these two samples overlap. In order to discern differences between these two samples, we have included a 3D PCA plot in the supplement of the revised manuscript (now Supplementary Figure S1). Please refer to lines 187-189 in the revised text.

Although the details of the PCA were included in the methods section, we agree that they should also be added to the figure caption. The figure caption has been revised to clarify that the PCA was constructed by first calculating the observed nucleotide frequency at each position in the SARS-CoV-2 genome for the wastewater sample, and then comparing the composite genotypes between two samples by summing the Yue and Clayton similarity index for each position in the genome. Please refer to lines 207-212 in the revised manuscript.

Moreover, regardless of what interpretation of genotype(s) is being computed for a dissimilarity score, it is difficult to believe that these two samples in particular would be perfectly identical on the first two PCs, given how many SNPs they have not-in-common, per figure 5 Clusters 3,2, and 1.

Since the PCA was constructed by calculating the sums of Yue and Clayton similarity indices for pairwise combinations of wastewater samples across all positions of the SARS-CoV-2 reference genomes, the contribution from mutations to the reference genome is small. Each position contributes a value between zero and one and there are 29,903 positions in the SARS-COV-2 genome. Since the PCA method has its limitations, we relied on multiple methods of comparison in the manuscript to compare the wastewater data.

***Other issues***

*Introduction*

The need to have Figure 5 and Figure S1 which only differ by the S1 having text labels on each row is odd? Figure S1 seems to be sufficient, and an idea would be to merge the two figures back into one, and in the body of the article. Thank you for this suggestion. Although a single figure with text labels would be our preference as well, unfortunately the text labels are illegible if the figure is shrunk to meet the allowable image dimensions for the journal (8.75 inches maximum height). Therefore, we request to keep both Figure 5 and Figure S1 in interest of preserving legibility for the reader.

Line 42-43: The statement that “reinfection with SARS-CoV-2 has been demonstrated to occur on college campuses in at least 16% of students” is interpreted incorrectly from Reference #8. The estimated reinfection rate in Reference 8 was 2.2% with 16% of the population susceptible to reinfection. This needs to be fixed. Thank you for this correction. We have corrected the sentence in lines 46-51 of the revised manuscript so that it is consistent with the reference.

*Figure 1*

Figure 1’s X axis makes sense in set notation, but probably not as what most readers will expect from a scientific figure. Would it be appropriate to replace the current x-axis label with the latter date of each given set’s range?

Thank you for this suggestion. We have removed the set notation and replaced the figure with a histogram where each bin is a two-day interval. Please see lines 80-831 for the revised Figure 1 caption.

*Figure 3*

Fig 3A is challenging to interpret. The gray dots along the axes aren’t easy to mentally place alongside the colorful diagonal dots. I advise the authors to rework this figure to 3 horizontal graphs, such as a top level with the gray UofSC-only sites, a middle with the colorful shared sites, and a bottom level with Columbia WWTP-only sites. Dot jitter could be used for legibility. Also, the Y-axis on Fig 3A uses the abbreviation “USC” not found elsewhere in the manuscript.

Thank you for these suggestions. We have changed the figure as recommended and modified the abbreviation to UofSC for consistency with the rest of the manuscript, as well as defined the acronym in the figure caption.

*Figure 4*

Axis labels and legends lack capitalization. Name the 4A legend something like “WWTP groups.”

Thank you for the suggestions. We have revised this figure to correct the capitalization. We chose to remove the legend title since the groups are evident based on names themselves.

*Linkage results and discussion*

The linkage discussion on divergence on p 13 is incomplete and could be made more clear. What is the significance or lack thereof of the disagreements? Also, there are some typos on line 293.

Thank you for these observations. We have removed the section regarding linkage analysis from the manuscript because we felt that it detracted from the main story of the manuscript and added little value. Instead, we incorporated greater detail into the cooccurrence analysis and a 20G outbreak on campus.

*Other minor notes*

On line 78, MGD has not been defined. Thank you for this comment. We have defined MGD as million gallons per day in the revised manuscript (lines 86-87).

On and around line 100, which native barcoding kit(s) were used? Expansion 1-12, 13-24, Expansion 96? Thank you for your comment. We used EXP-NBD196 (expansion 96). We have added this detail in lines 109-110 of the revised manuscript.

On line 104, unless all amplicons are exactly 400 bp, replace with “~400 bp.” Thank you for this suggestion. We have made the change.

On line 113, was the flow cell R9.4? If possible, more specific is better. Thank you for this suggestion. We have replaced R9 with R9.4.1, which is the exact flow cell that was used for all sequencing.

Citation 16 only has author initials. Thank you for noticing this. We have corrected the citation, although since the change was made using a reference manager, it is not reflected in the track changes.
